# Usefulness of immunohistochemical studies in diagnosing metachronous gallbladder and small intestinal metastases from lung cancer with gastrointestinal hemorrhage: a case report

**DOI:** 10.1186/s12957-015-0435-7

**Published:** 2015-02-18

**Authors:** Masayuki Tanaka, Minoru Kitago, Nobuyoshi Akiyama, Arifumi Iwamaru, Tatsuya Yamamoto, Fumio Suzuki, Taizo Hibi, Yuta Abe, Hiroshi Yagi, Masahiro Shinoda, Osamu Itano, Kentaro Ogata, Yuko Kitagawa

**Affiliations:** Department of Surgery, School of Medicine, Keio University, 35 Shinanomachi, Shinjuku, Tokyo, 160-8582 Japan; Department of Surgery, Kyosai Tachikawa Hospital, 4-2-22 Nishiki, Tachikawa, Tokyo, 190-8531 Japan; Department of Pathology, Kyosai Tachikawa Hospital, 4-2-22 Nishiki, Tachikawa, Tokyo, 190-8531 Japan

**Keywords:** Metachronous gallbladder and small intestinal metastases, Primary lung cancer, Gastrointestinal hemorrhage, Immunohistochemical staining

## Abstract

Isolated metachronous gastrointestinal metastases from advanced-stage lung cancer are rarely diagnosed on the basis of symptoms and resected. In this report, we present a case of resectable metachronous gallbladder and small intestinal metastases of lung cancer. An 86-year-old woman was treated for lung cancer with resection of the right inferior lobe. Five months after the surgery, she was re-admitted because of melena and anemia. Ultrasonography showed a gallbladder tumor with gastrointestinal hemorrhage, and laparoscopic-assisted cholecystectomy was subsequently performed. However, 2 months after this event, the patient presented again with melena and anemia and was diagnosed with a small intestinal tumor. Therefore, laparoscopic-assisted partial resection of the small intestine was performed. Immunohistochemical staining for thyroid transcription factor-1 and cytokeratin 7 confirmed that the two resected tumors were metachronous metastases of the primary lung cancer. The patient died of liver metastases 5 months after the last surgery. Our experience with this case suggests that surgical resection might not be curative but palliative for patients with isolated gallbladder and small intestinal metastases diagnosed on the basis of melena that is resistant to conservative treatment.

## Background

Although gastrointestinal metastases occasionally develop from advanced-stage primary lung cancer, isolated metachronous gastrointestinal metastases are rarely diagnosed on the basis of the symptoms and resected [1]. We recently experienced a case of metachronous gallbladder and small intestinal tumors presenting with gastrointestinal hemorrhage. The tumors were subsequently diagnosed as metastases from a primary lung cancer by immunohistochemical staining. We were able to perform successive laparoscopic-assisted palliative resection surgeries for the metastatic gallbladder and small intestinal tumors to control the patient’s anemia. Here, we present this case and discuss it with a review of the published literature.

## Case presentation

**Figure 1:**
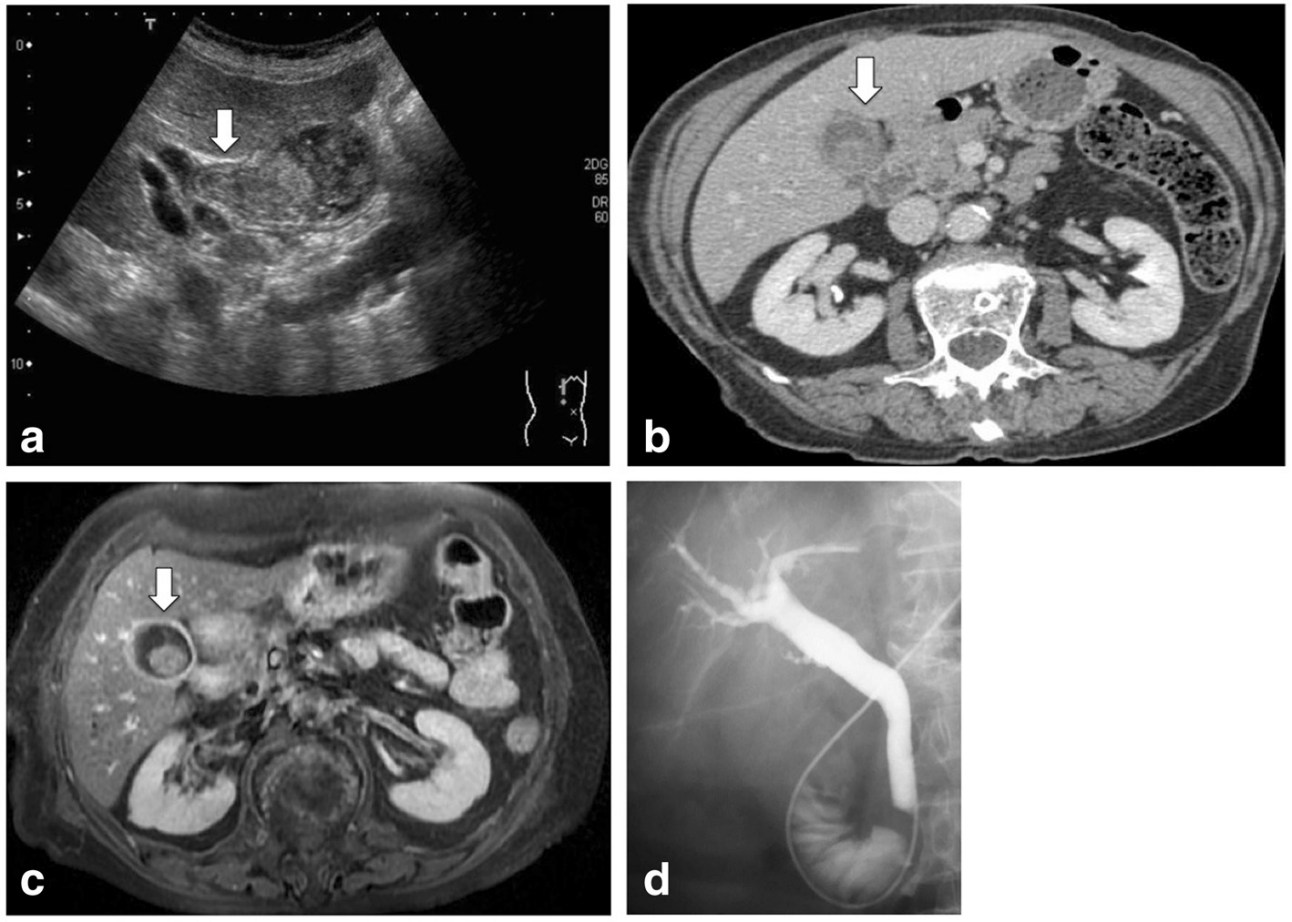
**The second preoperative imaging diagnoses. (a)** Ultrasonography, **(b)** computed tomography, and **(c)** magnetic resonance imaging (axial, T2-weighted image) findings. A 3.0 × 3.0-cm mass with enhancement was detected in the gallbladder; however, no lymph node enlargement or distant metastasis was observed. **(d)** Endoscopic retrograde cholangiography findings. Normal biliary tract without gallbladder filling was observed; however, hemobilia was recognized based on endoscopic nasal biliary drainage observations.

**Figure 2:**
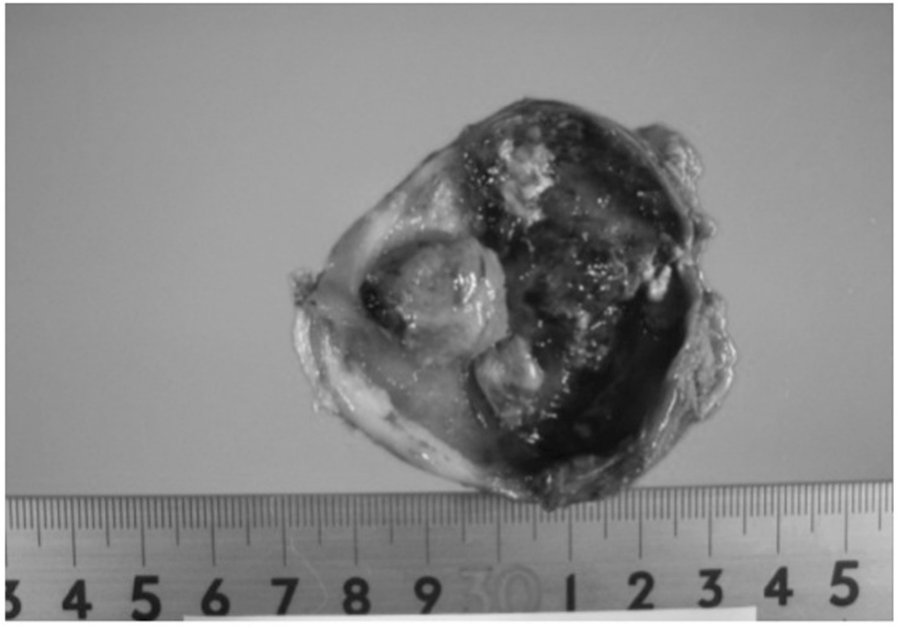
**Macroscopic findings of the resected gallbladder specimen.** The tumor measured 1.5 × 1.5 cm in size, with a clear margin, no invasion to other organs, and no metastasis.

An 86-year-old woman with a medical history of hypertension was treated for lung cancer with video-assisted thoracoscopic surgical resection of the right inferior lobe in September 2009. No distant metastasis was detected by preoperative positron emission tomography-computed tomography (PET-CT). Her histopathological diagnosis was adenocarcinoma with mixed solid adenocarcinoma and bronchioloalveolar carcinoma. The tumor’s Union for International Cancer Control (UICC) tumor-node-metastasis (TNM) classification was T2bN0M0 stage IIA. The patient received postoperative adjuvant chemotherapy with tegafur-uracil. However, 5 months after the surgery, in January 2010, she was urgently hospitalized because of melena and anemia. Laboratory data revealed her hemoglobin concentration to be 4.7 g/dL, which was lower than the 10.2 g/dL level measured a month prior to this event. No obvious source of bleeding was identified by both upper and lower gastrointestinal endoscopy. Ultrasonography revealed a gallbladder tumor (Figure 1a). A computed tomography (CT) scan of the area from the lungs to the pelvis and magnetic resonance imaging of the abdomen also revealed a 3.0-cm mass with enhancement in the gallbladder wall (Figure 1b,c). However, no lymph node enlargement or distant metastasis was observed. Endoscopic retrograde cholangiography revealed a normal biliary tract without gallbladder filling, but hemobilia was noted based on drainage from the endoscopic nasal biliary drainage tube (Figure 1d). This hemobilia was the deciding factor for our diagnosis of gastrointestinal hemorrhage due to a gallbladder tumor. In February 2010, after diagnostic laparoscopy to confirm the absence of other abdominal lesions, laparoscopic-assisted cholecystectomy was performed (Figure 2). The histological diagnosis of the resected gallbladder tumor was poorly differentiated adenocarcinoma, and its UICC TNM classification was T2aN0M0 stage IB. The patient’s anemia was improved, and she was discharged 10 days after the surgery, for a total of 48 days of hospitalization.

**Figure 3:**
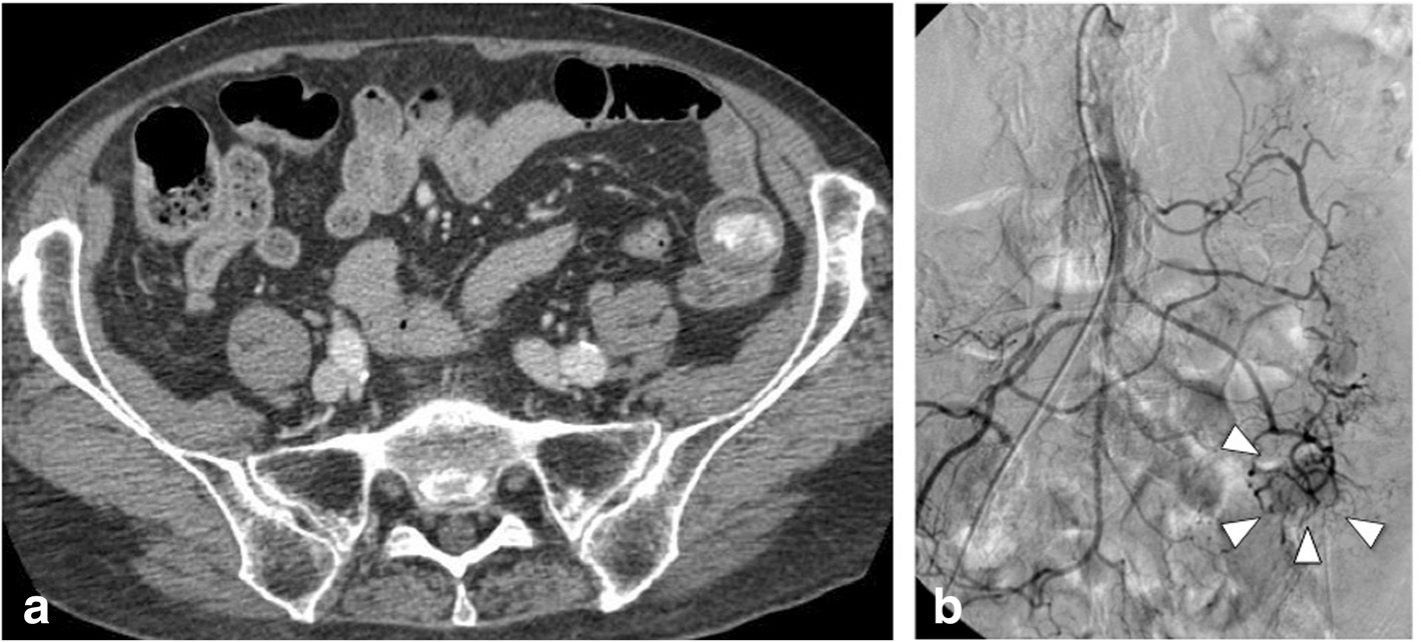
**The third preoperative imaging diagnoses. (a)** Enhanced computed tomography findings (axial). A small extravasation was observed in the small intestine with no tumor detected (arrow). **(b)** Angiography findings demonstrated tumor staining with no extravasation.

**Figure 4:**
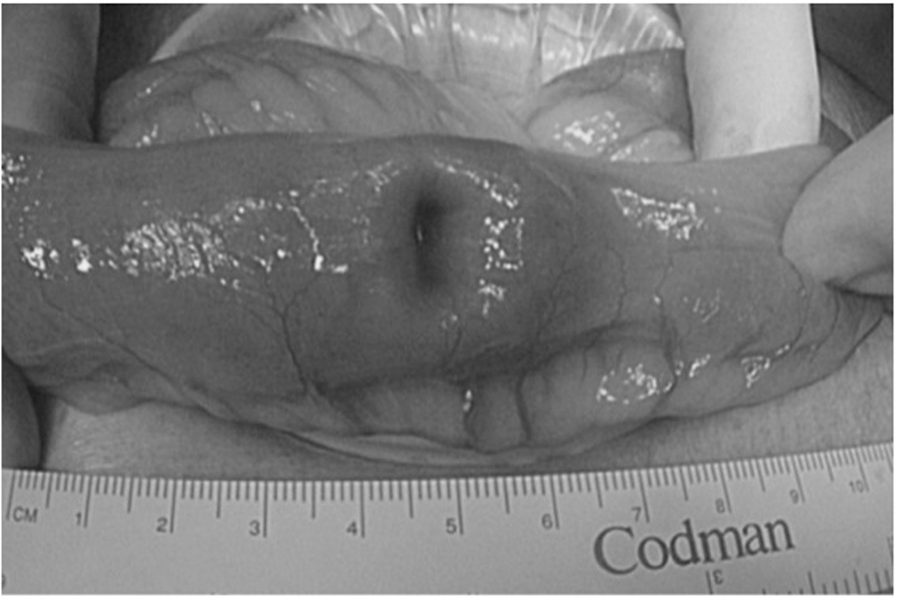
**Intraoperative findings of the small intestine.** The small intestinal serosa showed dimpling at approximately 140 cm from the Treitz ligament.

**Figure 5:**
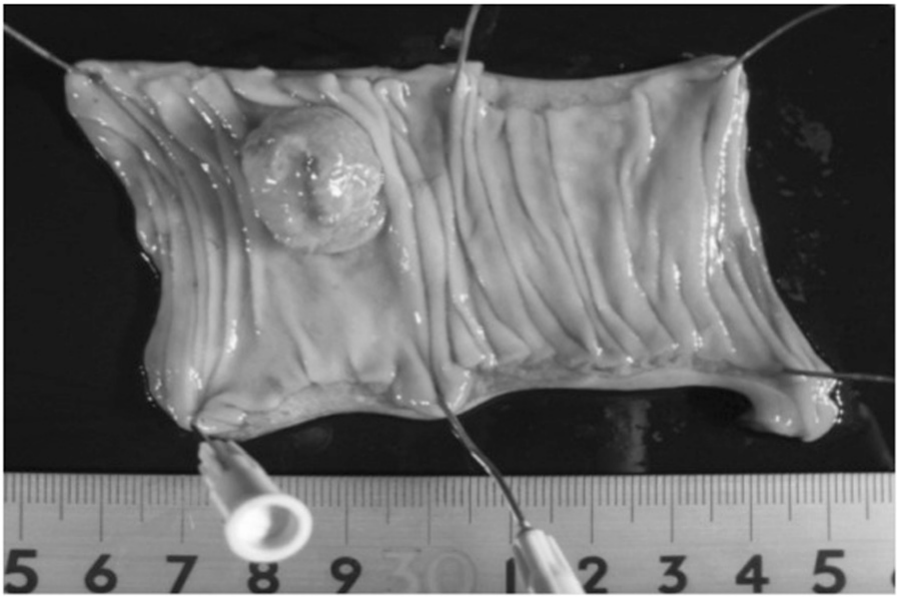
**Macroscopic findings of the resected intestinal specimen.** The tumor measured 2.0 × 1.5 cm in size, with a clear margin and no lymph node enlargement.

**Figure 6:**
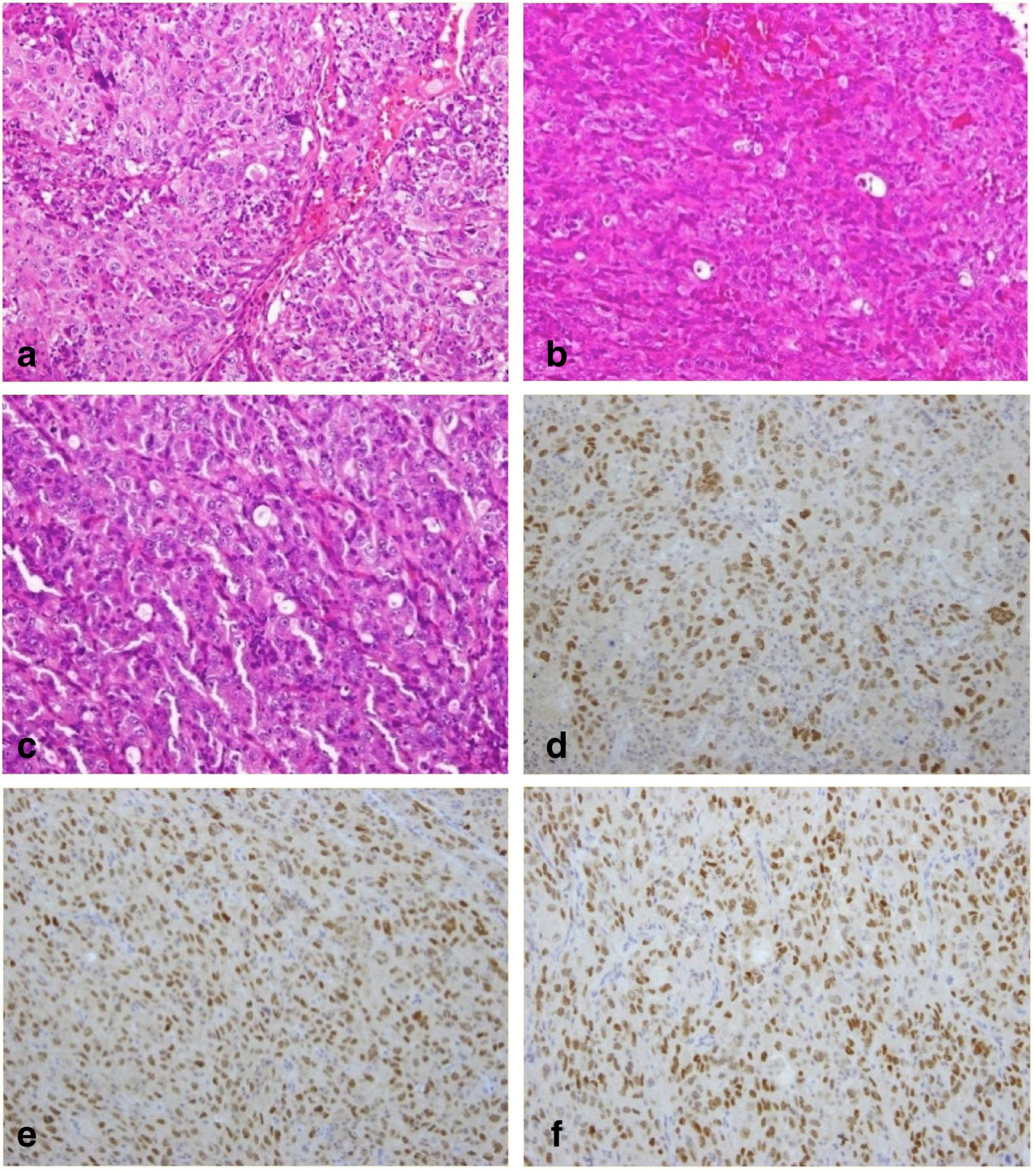
**Microscopic findings of the resected specimens.** Pathological features of the resected tumors. Histological examination of **(a)** the resected lung cancer, **(b)** the gallbladder tumor, and **(c)** the intestinal tumor showed poorly differentiated adenocarcinoma (hematoxylin and eosin stain, ×100). Immunohistochemistry of **(d)** the resected lung cancer, **(e)** the gallbladder tumor, and **(f)** the intestinal tumor showed that tumor cells were positive for thyroid transcription factor-1.

In April 2010, 2 months after the gallbladder surgery, the patient experienced melena and anemia again. No abnormality was detected on either upper or lower gastrointestinal endoscopy. However, a contrast-enhanced CT scan revealed slight extravasation in the small intestine, which was also observed on angiography (Figure 3a,b). Conservative therapy for 2 weeks was performed, but it did not improve the patient’s anemia. Laparoscopic surgery was therefore performed, and the small intestinal serosa was found to be dimpling at approximately 140 cm from the Treitz ligament (Figure 4). The whole small intestine was then examined via a small laparotomy. The tumor was only palpable at the dimpling site, which was considered to be the cause of the observed gastrointestinal hemorrhage. Subsequently, the small intestine was partially resected. The patient’s anemia improved once again, and she was discharged 13 days after the surgery, for a total of 34 days of hospitalization. The resected small intestinal tumor measured 2.0 × 1.5 cm in size, had a clear margin, and was of the intraluminal type (Figure 5). Microscopic examination of the tumor morphology by hematoxylin and eosin staining provided a histological diagnosis of a poorly differentiated adenocarcinoma (Figure 6a,b,c). An association between the primary lung cancer and the gallbladder and small intestinal tumors was suspected because of their similar histopathological classification as adenocarcinomas. Immunohistochemical studies indicated that cells from all three tumors were positive for thyroid transcription factor-1 (TTF-1) (Figure 6d,e,f) and cytokeratin 7 (CK7). Primary lung cancer with metachronous gallbladder and small intestinal metastases was therefore diagnosed. Although curative surgeries were performed, liver metastases were subsequently observed, and the patient died of progressive disease 5 months after the last surgery.

The most common distant metastasis site of lung cancer is the liver, and metastases in other intra-abdominal organs are very rarely detected before death [2]. Autopsy findings reported by Abrams *et al.* [3] indicated that involvement of the gallbladder and the small bowel was noted in approximately 1.9% of cases, each. Symptoms associated with gastrointestinal metastases such as abdominal pain, vomiting, anemia, and melena are often not present, and thus in most cases, metastatic tumors cannot be treated due to late discovery [4]. Even when the metastatic tumor is detected, the prognosis of lung cancer patients with gastrointestinal metastases is poor [2,5-7]. Additionally, as these metastases typically occur at advanced stages of disease, only palliative surgery is indicated. However, owing to the recently developed multidisciplinary therapy for lung cancer, the prognosis of such cases has been extended, and a few cases of isolated small intestinal metastases from a primary lung cancer have been reported to be curatively resected [1]. Such successful treatment outcomes of these small intestinal metastases may be attributable to the presence of only single intestinal lesions without abdominal lymph node involvement [1,8]. Reports of isolated gallbladder metastases from a primary lung cancer are even less common [9,10], and no report is available on the successful resection of both isolated small intestinal and gallbladder metastases via minimally invasive surgery.

TTF-1 is a very sensitive and specific marker of the pulmonary origin of an adenocarcinoma, if a thyroid origin has been excluded [11-13]. Therefore, instead of a lung-specific marker, TTF-1 can be used to discriminate between metastases from a primary lung cancer and primary gallbladder cancer [14,15]. CK7 is also a common marker for primary lung adenocarcinoma as is expressed in 100% of cases; however, the specificity of CK7 is not as high as it is also expressed in 27% of non-pulmonary adenocarcinomas [15,16]. Metastatic gallbladder tumors are very rare, and differentiating between primary and metastatic gallbladder cancer is challenging. Therefore, in the present case, we were able to diagnose the gallbladder tumor as metastasis from the primary lung cancer by immunohistochemical staining for TTF-1 and CK7.

In most cases, symptoms are not observed in the early stages [4]. As a result, patients should be carefully followed up for gastrointestinal metastases with imaging studies, including both contrast-enhanced CT and PET-CT, the combined use of which has been reported to improve sensitivity and specificity and help in early detection, leading to curative resection and better prognosis [17-20]. Usually, it is assessed by different authors that 4 months of the time from diagnosis of gastrointestinal metastasis to death is very short [4,21].

If a metastatic tumor from the primary lung cancer is isolated and the primary disease is well controlled, resection should be the first choice. However, surgical intervention might not be indicated in cases of multiple metastases and in case in which prognosis cannot be improved. Therefore, careful examination with imaging studies including chest CT, contrast-enhanced CT of the abdominopelvic region, or PET-CT should be conducted before a decision regarding surgery can be made [22,23].

In the present case, we could not perform PET-CT, but we examined carefully by using other imaging studies, including enhanced CT, upper and lower gastrointestinal endoscopy, endoscopic retrograde cholangiography, and angiography, to diagnose the respective isolated metachronous metastases.

Although the gallbladder and small intestinal metastases could have been part of systemic disease, no other lesions were detected by either preoperative imaging studies or intraoperative examination. Therefore, each tumor was diagnosed as an isolated metastasis and resected by a minimally invasive procedure to resolve the patient’s gastrointestinal hemorrhage and anemia that were uncontrollable with conservative treatment. Although such resection procedures might not be curative, these procedures were worth performing to improve the patient’s quality of life [5,8]. To our knowledge, this is the first report of isolated metachronous gallbladder and small intestinal metastases of lung cancer with symptoms which were resectable.

## Conclusions

We encountered a case of isolated resectable metachronous gallbladder and small intestinal metastases from a primary lung cancer, which were detected by gastrointestinal hemorrhage. Immunohistochemical staining was useful in establishing a connection with the primary cancer.

## Consent

Written informed consent was obtained from the patient for publication of this case report and any accompanying images. A copy of the written consent is available for review by the Editor-in-Chief of this journal.

## Abbreviations

CK7: Cytokeratin 7
PET-CT: positron emission tomography-computed tomography
CT: computed tomography
TTF-1: thyroid transcription factor-1

## References

[1] Kim MS, Kook EH, Ahn SH, Jeon SY, Yoon JH, Han MS (2009). Gastrointestinal metastasis of lung cancer with special emphasis on a long-term survivor after operation. J Cancer Res Clin Oncol.

[2] Stenbygaard LE, Sørensen JB (1999). Small bowel metastases in non-small cell lung cancer. Lung Cancer.

[3] Song Y, Li M, Shan J, Ye X, Tang S, Fang X (2012). Acute small bowel obstruction: a rare initial presentation for the metastasis of the large-cell carcinoma of the lung. World J Surg Oncol.

[4] Yang CJ, Hwang JJ, Kang WY, Chong IW, Wang TH, Sheu CC (2006). Gastro-intestinal metastasis of primary lung carcinoma: clinical presentations and outcome. Lung Cancer.

[5] Nishizawa Y, Kobayashi A, Saito N, Nagai K, Sugito M, Ito M (2012). Surgical management of small bowel metastases from primary carcinoma of the lung. Surg Today.

[6] Kanemoto K, Kurishima K, Ishikawa H, Shiotani S, Satoh H, Ohtsuka M (2006). Small intestinal metastasis from small cell lung cancer. Intern Med.

[7] Sakai H, Egi H, Hinoi T, Tokunaga M, Kawaguchi Y, Shinomura M (2012). Primary lung cancer presenting with metastasis to the colon: a case report. World J Surg Oncol.

[8] Berger A, Cellier C, Daniel C, Kron C, Riquet M, Barbier JP (1999). Small bowel metastases from primary carcinoma of the lung: clinical findings and outcome. Am J Gastroenterol.

[9] Nassenstein K, Kissler M (2004). Gallbladder metastasis of non-small cell lung cancer. Onkologie.

[10] Jeong YS, Han HS, Lim SN, Kim MJ, Han JH, Kang MH (2012). Gallbladder metastasis of non-small cell lung cancer presenting as acute cholecystitis. Chin J Cancer Res.

[11] Mukhopadhyay S, Katzenstein AL (2012). Comparison of monoclonal napsin A, polyclonal napsin A, and TTF-1 for determining lung origin in metastatic adenocarcinomas. Am J Clin Pathol.

[12] Jagirdar J (2008). Application of immunohistochemistry to the diagnosis of primary and metastatic carcinoma to the lung. Arch Pathol Lab Med.

[13] Jan IS, Chung PF, Weng MH, Huang MS, Lee YT, Kuo SH (2006). Utility of thyroid transcription factor-1 expression in the differential diagnosis of metastatic adenocarcinoma of serous effusion specimens prepared using the cell transfer technique. J Formos Med Assoc.

[14] Jerome MV, Mazieres J, Groussard O, Garcia O, Berjaud J, Dahan M (2004). Expression of TTF-1 and cytokeratins in primary and secondary epithelial lung tumours: correlation with histological type and grade. Histopathology.

[15] Montezuma D, Azevedo R, Lopes P, Vieira R, Cunha AL, Henrique R (2013). A panel of four immunohistochemical markers (CK7, CK20, TTF-1, and p63) allows accurate diagnosis of primary and metastatic lung carcinoma on biopsy specimens. Virchows Arch.

[16] Park YJ, Kim KY, Park JY, Cho JS, Kim Y, Shin SH (2013). Generalized peritonitis arising from small bowel metastasis in a lung cancer patient. J Korean Surg Soc.

[17] Huang YM, Hsieh TY, Chen JR, Chien HP, Chang PH, Wang CH (2012). Gastric and colonic metastases from primary lung adenocarcinoma: a case report and review of the literature. Oncol Lett.

[18] Pieterman RM, van Putten JW, Meuzelaar JJ, Mooyaart EL, Vaalburg W, Koëter GH (2000). Preoperative staging of non-small-cell lung cancer with positron-emission tomography. N Engl J Med.

[19] Gould MK, Kuschner WG, Rydzak CE, Maclean CC, Demas AN, Shigemitsu H (2003). Test performance of positron emission tomography and computed tomography for mediastinal staging in patients with non-small-cell lung cancer: a meta-analysis. Ann Intern Med.

[20] Loukeri AA, Kampolis CF, Ntokou A, Tsoukalas G, Syrigos K (2015). Metachronous and synchronous primary lung cancers: diagnostic aspects, surgical treatment, and prognosis. Clin Lung Cancer.

[21] Bugiantella W, Cavazzoni E, Graziosi L, Valiani S, Franceschini MS, Donini A (2011). Small bosel metastasis from lung cancer: apossible cause of acute abdomen. Case report and literature review. G Chir.

[22] Lee JW, Kim SK, Park JW, Lee HS (2010). Unexpected small bowel intussusception caused by lung cancer metastasis on ^18^F-fluorodeoxyglucose PET-CT. Ann Thorac Surg.

[23] Akamatsu H, Tsuya A, Kaira K, Nakamura Y, Naito T, Murakami H (2010). Intestinal metastasis from non-small-cell lung cancer initially detected by ^18^F-fluorodeoxyglucose positron emission tomography. Jpn J Radiol.

